# What is community pharmacists’ level of comfort and interest in managing patients with or at risk of major neurocognitive disorders?

**DOI:** 10.1177/17151635221128552

**Published:** 2022-10-13

**Authors:** Khaarthikaa Murugesu, Olivier Massé, Anne Maheu, Line Guénette

**Affiliations:** Faculté de pharmacie, Université de Montréal, Montréal; Faculté de pharmacie, Université de Montréal, Montréal; Centre intégré universitaire de santé et de services sociaux (CIUSSS) du Nord-de-l’île-de-Montréal, Montréal; Faculté de pharmacie, Université de Montréal, Montréal; Centre intégré universitaire de santé et de services sociaux (CIUSSS) du Nord-de-l’île-de-Montréal, Montréal; Faculté de pharmacie, Université Laval, Québec; Centre de Recherche du CHU de Québec, Axe Santé des Populations et Pratiques Optimales en Santé, Québec, Québec

## Introduction

Major neurocognitive disorders (MNCDs) have been forecasted to almost triple globally to 152 million people by 2050.^1^ In MNCDs, the brain is impacted so that cognitive processes, such as memory, language, organizational abilities, planning and judgment, gradually deteriorate.^2^ With considerable damage, a person’s daily life is impaired, decreasing autonomy and further resulting in requiring assistance with everyday tasks.^3^

The Plan Alzheimer du Québec (PAQ) was created in 2013 based on an existing provincial health and social services framework to allow optimal care of patients with cognitive impairments.^4^ This initiative equips the Family Medicine Groups (FMGs) of the province to manage patients with cognitive disorders.^5^ FMGs are collaborative and interdisciplinary primary care clinics consisting of family physicians, nurses and, more recently, social workers and pharmacists.^6^ These professionals provide care and offer resources that aim to sustain the autonomy of patients affected by MNCDs.

In line with the PAQ, the Projet GPS is an ongoing pragmatic controlled study funded by the “Fonds de recherche du Québec-Santé.” It aims to measure the impact of pharmacists’ contributions to FMGs regarding patients who either have MNCDs or are undergoing cognitive assessment.^7^ FMG pharmacists’ interventions focus on optimizing a patient’s medications.^8^ They aim to improve quality of life by managing prescriptions and deprescribing those that are suboptimal.^9-11^ Medication optimization is crucial for older persons to remove medications that may exacerbate their cognitive disorders.^12^ Therefore, as a living laboratory-type intervention, the Projet GPS will enable us to explore additional elements of MNCD patients’ journeys, including connecting with community pharmacy.

One of a patient’s first contact points with the health care system is the community pharmacy. From a study in 2016, it was seen that about 25% of Canadian seniors typically take 10 or more prescription drugs per year, making community pharmacists an essential player on their health care team.^13^ It has been established that the elderly with cognitive problems are more prone to increased risk of adverse drug events.^14^ With the notable increase in chronic illnesses within the elderly community, pharmacists are well placed to facilitate the early detection of those who present signs of cognitive impairments and support their health system navigation.^14,15^

Although community pharmacists play a distinct interactive role with patients, which could be advantageous in optimizing the pharmacotherapy of those with or at risk of MNCDs and flagging patients who require medical attention to their FMGs, little is known about their level of comfort and willingness to engage in these activities. Therefore, this project’s goal was to survey pharmacists (both licensed and final-year pharmacy students) practising or wishing to practise in community pharmacy settings about their role among the elderly with or at risk of MNCDs.

## Methods

### Participant population

We invited licensed pharmacists and fourth-year pharmacy students in Québec to participate in a web-based survey. The sole inclusion criterion was to be primarily practising in a community pharmacy setting or, for students, wishing to do so.

### Questionnaire

A fourth-year pharmacy student doing an internship under the supervision of 2 of the GPS researchers designed the online questionnaire. A pharmacist and 2 pharmacy students pretested the questions to ensure their functionality and clarity. The questionnaire and all correspondences were in French. We emailed a Google Forms link containing the study questions to community pharmacists practising in the Centre intégré universitaire de santé et de services sociaux (CIUSSS) du Nord-de-l’île-de-Montréal (NIM) territory, as the Projet GPS is being implemented in this area. A CIUSSS is responsible for providing health care services to its population. The link to the questionnaire was also made available through Facebook on the 2017-2021 pharmacy student cohort of the Université de Montréal group and on the Ordre des pharmaciens du Québec page. The questionnaire was accessible from April 16 to 20, 2021. As mentioned to participants, we regarded questionnaire completion as consent and authorization of results publication.

The survey consisted of 15 questions grouped into 5 sections: 1) general overview of participant population (*n* = 4), 2) recognition of the signs and symptoms associated with MNCDs (*n* = 3), 3) pharmacotherapy optimization (*n* = 3), 4) pharmacotherapy monitoring (*n* = 2) and 5) evaluation of needs (*n* = 3). Most of the questions were assessed with a 5-point Likert scale.

### Analysis

We conducted a descriptive analysis. We computed the frequency of each response chosen by the respondents. Proportions of respondents were calculated for each question. Median and mean scores of the Likert scale were calculated by question. We also computed the mean of the mean scores by theme. These are presented in Table 1.

**Table 1:** Community pharmacists’ interest and comfort with patients with or at risk for MNCDs (*N* = 107)

	Score on the Likert scale							
Themes and questions asked	1 (not at all) (%)	2 (%)	3 (%)	4 (%)	5 (extremely) (%)	Median score	Mean score	SD
Interest of pharmacists^*^ **4.06** General participants’ interest in this population								
1. How interested are you in patients with or at risk for MNCDs?	0	7.5	22.4	50.5	19.6	4	3.82	0.83
Recognition of the signs and symptoms associated with MNCDs								
2. How interested are you in intervening and referring more often (to their FMG or clinic) a patient suspected of having MNCDs, for a complete cognitive evaluation?	0	4.7	14	48.6	32.7	4	4.09	0.81
Pharmacotherapy optimization								
3. How interested are you in working more often with patients and prescribers to optimize drug therapies for patients with or at risk for MNCDs?	0	2.8	10.3	46.7	40.2	4	4.24	0.75
Pharmacotherapy monitoring								
4. How interested are you in following up with patients taking medications pertaining to MNCDs or BPSD?	0.9	2.8	13.1	55.1	28	4	4.07	0.78
**Comfort of pharmacists**^†^** 3.34** Recognition of the signs and symptoms associated with MNCDs								
5. How comfortable are you in identifying the signs and symptoms characteristic of MNCDs?	3.7	17.8	41.4	31.8	5.6	3	3.18	0.92
6. How comfortable are you with intervening and referring (to their FMG or clinic) a patient suspected of having MNCDs, for a complete cognitive evaluation?	13.1	32.7	36.4	13.1	4.7	3	2.64	1.02
Pharmacotherapy optimization								
7. How comfortable are you in analyzing the pharmacological profile of patients with or at risk for MNCDs?	0	8.4	22.4	56.1	13.1	4	3.74	0.79
8. How comfortable are you in intervening with patients and prescribers to optimize pharmacotherapies (e.g., pharmaceutical opinion, benzodiazepine deprescribing, reduction of anticholinergic load) of patients with or at risk of MNCDs?	0	9.3	25.2	41.1	24.3	4	3.80	0.92
Pharmacotherapy monitoring								
9. For the following drugs used in MNCDs or BPSD (e.g., aggressiveness, anxiety, apathy, insomnia), how comfortable are you with their clinical follow-up (efficacy, safety, adherence)?								
a) AchEI	3.7	12.1	30.8	44.9	8.4	4	3.46	0.92
b) Memantine	6.5	26.2	38.3	27.1	1.9	3	2.92	0.93
c) Antipsychotics	3.7	10.3	37.4	37.4	11.2	3	3.42	0.95
d) Antidepressants	1.9	4.7	20.6	49.5	23.4	4	3.88	0.89
e) Sedative hypnotics	1.9	4.7	24.3	51.4	17.8	4	3.79	0.86

AchEI, acetylcholinesterase inhibitors; BPSD, behavioural and psychological symptoms of dementia; FMG, family medicine group; MNCDs, major neurocognitive disorders; SD, standard deviation.

* Questions 1-4: Level of interest varied from 1 = not at all interested, 2 = slightly interested, 3 = neutral, 4 = interested, 5 = extremely interested.

† Questions 5-9: Level of comfort varied from 1 = not at all comfortable, 2 = slightly comfortable, 3 = neutral, 4 = comfortable, 5 = extremely comfortable.

**Table 2:** Evaluation of pharmacists’ needs on training and resources (*N* = 107)

Questions	Response	%
Support in the management of patients		
Which of the following options do you think you need further training to better participate in the management of patients with or at risk for MNCDs? Select one or more options.	Knowledge of the resources in your region to refer a patient if necessary	88.8
	Identification of concerns requiring risk stratification and referral to resources (e.g., 911, social work resources, home care, Elder Abuse helpline, etc.)	70.1
	Communication with the patient after identifying signs and symptoms of MNCDs or BPSD	58.9
	Follow-up of treatments for BPSD (e.g., antipsychotics)	54.2
	Monitoring of acetylcholinesterase inhibitors or memantine	53.3
	Identification of signs and symptoms attributable to MNCDs or BPSD	47.7
	Intervention with the patient and the prescriber to optimize pharmacotherapies (e.g., pharmaceutical opinion, deprescribing benzodiazepines)	36.4
	Analysis of pharmacotherapies (e.g., potentially inappropriate medications)	34.6
Other (with the option to elaborate):	“Resources and collaboration among parties involved” “Legislation and potential breach of confidentiality”	
Resources to better support the patients		
Which of the following would allow you to improve the management of patients with MNCDs, identifying and revising the pharmacological profile and clinical monitoring of their pharmacotherapy? Select one or more options.	A pharmacist-friendly guide to the care regarding this population	80.4
	A directory with the most useful resources in different regions	79.4
	A toolbox of relevant resources	66.4
	A self-training session summarizing the care concerning this population	53.3
	A PowerPoint training session summarizing the care concerning this population	41.1
	Case studies of patients met in community pharmacy	35.5
	Round table discussions on patient cases that you have encountered in your practice and those which have been difficult	10.3
Other (with the option to elaborate):	“PowerPoint with resources, training modules.”	

BPSD, behavioural and psychological symptoms of dementia; MNCDs, major neurocognitive disorders.

## Results

A total of 107 respondents agreed to participate and fully completed the web-based questionnaire. Among them, 66.4% were licensed pharmacists practising in community pharmacies, whereas the rest (33.6%) were fourth-year pharmacy students with the intention of continuing their practice in a community pharmacy setting. Of those who participated, 20.6% practised or planned to practise in the CIUSSS du NIM territory.

Table 1 presents respondents’ interest and comfort with patients with or at risk for MNCDs. The mean score for questions about interests (questions 1-4) was 4.06 (where a score of 5 means “extremely”), while it was 3.34 for questions about comfort level (questions 5-8).

### General overview of participants’ interest in this population

Most of the participants (70.1%) were either “extremely interested” or “interested” in working with patients with or at risk of MNCDs. Although participants expressed interest in helping patients with or at risk of MNCDs, 61.7% admitted that they had “very poor” or “poor” knowledge of the resources available in this domain.

### Recognition of the signs and symptoms associated with MNCDs

As presented in Table 1, many participants (81.3%) were interested (“extremely interested” or “interested”) in intervening and referring a patient for a complete cognitive evaluation. However, only a fifth (17.8%) felt comfortable (“extremely comfortable” or “comfortable”) in doing so. In contrast, a third of the participants (37.4%) said they were comfortable identifying the signs and symptoms associated with MNCDs.

### Pharmacotherapy optimization

With regards to working more often with patients and prescribers to optimize pharmacotherapies of patients with or at risk of MNCDs, a substantial proportion of participants (86.9%) were interested (“extremely interested” or “interested”) and 65.4% of the participants said they were comfortable (“extremely comfortable” or “comfortable”).

### Pharmacotherapy monitoring

Most participants (83.1%) were interested (“extremely interested” or “interested”) in following up with patients taking medications for MNCDs or behavioural and psychological symptoms of dementia (BPSD). We asked pharmacists and students to rank their comfort level concerning clinical follow-ups of 5 classes of medications frequently used in patients with MNCDs. Respondents expressed a higher level of comfort (“extremely comfortable” or “comfortable”) with antidepressants (72.9%) and sedative-hypnotics (69.2%) than with acetylcholinesterase inhibitors (AChEI) (53.3%), antipsychotics (48.6%) and memantine (29%) follow-ups.

### Evaluation of needs

We gave participants a list of activities, skills or knowledge that they could select to indicate what training they thought they needed. As presented in Table 2, the 2 most chosen elements were 1) risk stratification and referral to resources (70.1%) and 2) communication with patients after detection of signs of cognitive impairment (58.9%). Participants were less likely to choose elements related to the analysis and optimization of pharmacotherapy (36.4% and 34.6%, respectively).

## Discussion

From the survey results, it is apparent that community pharmacists feel comfortable with their knowledge of pharmacotherapy and are interested in being actively involved in the care of patients with or at risk of MNCDs. Although the respondents expressed a high interest, their lack of comfort in identifying the early signs of cognitive disorders or in intervening/referring a patient with suspected MNCDs hinders optimal pharmacological treatments and early referrals to the appropriate professionals. The participants admitted to having limited knowledge of the resources available to patients with or at risk of MNCDs. In addition, they reported that their communication skills with this population need to be improved.

Before this study, a literature review demonstrated that most studies were on pharmacists’ knowledge of pharmacotherapy to treat MNCDs instead of their level of interest in and comfort with this population.^16-18^ However, as shown in a study about factors influencing pharmacists’ adoption of new practices,^19^ knowledge is not the sole element in the equation. The complexity associated with these cases, the type of practice setting and the relationship with the patient’ physician are all factors that can affect a pharmacist’s adoption of new activities.^19^ A study similar to ours done in Northern Ireland demonstrates that, even when having the interest to intervene in pharmacotherapies, pharmacists require more training to be comfortable doing so.^20^ We also suspect they require more experience and that their practice settings need some transformation.

One of the strengths of the present study is its objective’s originality, which does not aim to measure the knowledge of participants but rather their interest and level of confidence. To our knowledge, it is one of the first studies on this question. The questionnaire was developed by 2 pharmacists and a fourth-year pharmacy student, as well as pretested by a pharmacist and 2 pharmacy students. This ensured questions were pertinent and well understood, a requisite for valid results. However, we must acknowledge some limitations and call for caution in interpreting the results. Due to its short duration, our survey sample size (*N* = 107) was relatively small and might not be representative of all community pharmacists of the province of Québec. Although we were interested in pharmacists’ level of comfort and interest in managing patients with or at risk of major neurocognitive disorders in community pharmacies, a third of our population were fourth-year pharmacy students with limited practice experience. Moreover, participants were not randomly selected but were instead limited to interested pharmacists and students, potentially allowing selection and information bias. Lastly, although not suspected, the inability to verify the possibility of duplicate answers is a limitation as well.

Our study supports the importance of developing and publicizing tools adapted to community pharmacists and better educating pharmacists and pharmacy students in managing patients with or at risk of MNCDs. In addition, we identified potential solutions to help improve community pharmacists’ practice when intervening with patients and optimizing pharmacotherapy. We developed tools that could aid in closing the gap that community pharmacists feel with their knowledge and comfort in intervening with patients with or at risk of MNCDs. A decision-making algorithm to help community pharmacists identify specific elements suggestive of MNCDs and refer patients to available resources was created and is available at https://www.ciusssnordmtl.ca/zone-des-professionnels/pharmaciens/. It can be accessed under the heading “Aide à la décision pour la clientèle avec ou à risque de trouble neurocognitif majeur.”^21^ Additionally, a directory of local resources (e.g., 211, social workers, Elder Abuse Helpline) adapted to the needs of the CIUSSS du NIM pharmacists was developed and is also available on their website.^22^ Both tools have been shared with pharmacists, and more active dissemination is being explored.

Last, our results suggest it would be beneficial to create video modules demonstrative of common scenarios and effective communication techniques pharmacists could use to ease their future interventions. An MNCD-specific self-learning module is available for FMG pharmacists participating in the Projet GPS. We plan broader dissemination after the completion of this study. Combining these measures would further educate pharmacists on early interventions concerning MNCDs.

One of the PAQ’s objectives for its phase 3 initiative (2022-2024) is to extend its outreach to community pharmacists who could help support patients with or at risk of MNCDs.^5,23,24^ One such element includes early referral to the medical team when warranted. It is believed that the FMG pharmacists could serve as an intermediate between community pharmacists and family doctors. Facilitating early detection of patients at risk of MNCDs could allow for better outcomes because of earlier consultations and care.

## Conclusion

Although community pharmacists’ interest in this field is high and relevant, there is a knowledge gap preventing them from being comfortable in intervening with patients with or at risk of MNCDs. Management of these patients is thus challenging due to the disorder’s complexity, which is only furthered by the lack of resources available for patients and pharmacists. Therefore, the development and greater accessibility of valuable tools and training would better equip community pharmacists to use their existing knowledge and improve their comfort in managing patients with or at risk of MNCDs. ■
